# Veterinary Medical Association of New York County

**Published:** 1900-05

**Authors:** Robert W. Ellis

**Affiliations:** Secretary


### SOCIETY PROCEEDINGS.

### VETERINARY MEDICAL ASSOCIATION OF NEW YORK COUNTY.

THE regular monthly meeting was called to order March 7th, at 8.30 o'clock, by President Robertson. After roll-call, reading of minutes of previous meeting, etc., by the Secretary, and the report of the Board of Censors had been heard through Chairman Clayton, a paper on "Docking" was presented by Dr. Ryder. This paper, which was carefully prepared so as to touch upon all phases of the operation, was productive of a very animated and interesting discussion. A vote of thanks was offered the essayist from the Association through Dr. O'Shea, and seconded by Dr. Bell. Dr. O'Shea reported progress from the Legislative Committee. Dr. Bretherton, chairman of the Prosecuting Committee, had no report to offer at this time, but desired a communication to be read by the Secretary relative to an advertisement from a Brooklyn non-graduate. After the reading the President directed the matter to be referred to the Brooklyn member of the Prosecuting Committee, Dr. Goubeaud. Dr. Bell, chairman of Ways and Means Committee, reported a paper for the April meeting by Dr. Hanson. Moved and seconded, that the reports of the several committees be accepted; carried. Moved and seconded, that the meeting adjourn; carried.

The meeting was called to order April 11th, at the usual hour, in the lecture-room of the New York American Veterinary College, President Robertson presiding. Following the usual business proceedings of the Association, Dr. H. D. Hanson gave an extemporaneous discourse upon "What Constitutes a Spavin in an Examination for Soundness." The doctor stated that his principal object in offering his remarks upon this subject was to provoke a discussion, and in this he was decidedly successful, this practical every-day subject seeming to appeal to every one present. Dr. Delaney, the Association wit, stated that some man from New Jersey had a few days previously found a horse in one of our New York horse marts with three spavins on one leg. Prof. Schwarzkopf asked whether saddle horses were more subject to spavin than driving horses. The essayist stated that his practice brought him in contact with too few saddle horses to be able to draw a conclusion. Dr. Ackerman stated, in reply to Prof. Schwarzkopf's question, that his connection for a number of years with a riding academy as its veterinarian had taught him that this condition was not prevalent among that class of horses, he having treated but very few cases of spavin in the academy.

Legislative Committee—Dr. O'Shea, chairman, reported that his committee would be unable to report until the Legislature adjourns, but at the instigation of Prof. Schwarzkopf offered the following resolution, to be sent to Senators Platt and Depew, Washington, D.C.:

Whereas, the United States is the only civilized government in the world whose army has no veterinary corps or properly equipped veterinarians for the care of its public animals and for the inspection of meat consumed by its soldiers;

Therefore, we, the Veterinary Medical Association of New York County, do most respectfully ask you, our representatives in the Senate, to request the Secretary of War and your colleagues in the Senate to establish a veterinary corps in the United States army worthy of our great country.

The above to be signed by the President and Secretary of the Association, sealed with the Association's seal, and forwarded to Senators Platt and Depew.

The Committee on Prosecution having nothing further to report, President Robertson reported, in the absence of the Ways and Means Committee, that Dr. Lellman would present some specimens at the May meeting. Moved and seconded, that the meeting adjourn.

ROBERT W. ELLIS, D.V.S.,
Secretary.

